# Guanidinium (aqua-2κ*O*)(4-hydr­oxy-6-carboxy­pyridine-2-carboxyl­ato-2κ^3^ *O* ^2^,*N*,*O* ^6^)(μ-4-hydroxy­pyridine-2,6-dicarboxyl­ato-1:2κ^4^ *O* ^2^,*N*,*O* ^6^:*O* ^2^)(4-hydroxy­pyridine-2,6-dicarboxyl­ato-1κ^3^ *O* ^2^,*N*,*O* ^6^)dizincate(II) dihydrate

**DOI:** 10.1107/S1600536808003930

**Published:** 2008-02-13

**Authors:** Hossein Aghabozorg, Somayeh Saadaty, Elham Motyeian, Mohammad Ghadermazi, Faranak Manteghi

**Affiliations:** a Faculty of Chemistry, Tarbiat Moallem University, 49 Mofateh Ave., 15614 Tehran, Iran; b Department of Chemistry, Islamic Azad University, North Tehran Branch, Tehran, Iran; c Department of Chemistry, Faculty of Science, Payame Noor University, Qom Center, Qom, Iran; d Department of Chemistry, Faculty of Science, University of Kurdistan, Sanandaj, Iran; e Faculty of Chemistry, Iran University of Science and Technology, Tehran, Iran

## Abstract

The title compound, (CH_6_N_3_)[Zn_2_(C_7_H_3_NO_5_)_2_(C_7_H_4_NO_5_)(H_2_O)]·2H_2_O, has an anionic binuclear complex of Zn^II^ balanced with a guanidinium cation. There are two uncoord­inated water mol­ecules in the structure. The asymmetric unit of the compound has two different coordination types (the coordination of Zn1 is distorted trigonal-bipyramidal, while that of Zn2 is distorted octahedral) of Zn^II^ in the crystal structure that are bridged to each other *via* one hypydc^2−^ group (hypydcH_2_ is 4-hydroxy­pyridine-2,6-dicarboxylic acid). A variety of inter­molecular O—H⋯O, N—H⋯O and C—H⋯O hydrogen bonds involving water mol­ecules, cations and anions, and also a weak π–π inter­action [3.798 (1) Å], are responsible for extending the structure into a three-dimensional network.

## Related literature

For related literature, see: Moghimi, Aghabozorg, Sheshmani, *et al.* (2005 ▶); Moghimi, Aghabozorg, Soleimannejad *et al.* (2005 ▶); Aghabozorg *et al.* (2008 ▶); Ranjbar *et al.* (2002 ▶); Sharif *et al.* (2007 ▶).
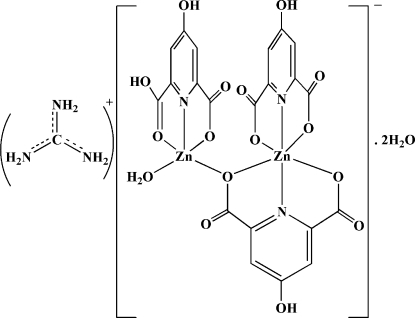


## Experimental

### 

#### Crystal data

(CH_6_N_3_)[Zn_2_(C_7_H_3_NO_5_)_2_(C_7_H_4_NO_5_)(H_2_O)]·2H_2_O
*M* *_r_* = 789.20
Triclinic, 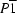

*a* = 9.1077 (12) Å
*b* = 9.2900 (12) Å
*c* = 16.347 (3) Å
α = 96.018 (4)°
β = 99.229 (4)°
γ = 91.760 (7)°
*V* = 1356.1 (3) Å^3^
*Z* = 2
Mo *K*α radiation
μ = 1.87 mm^−1^
*T* = 100 (2) K
0.21 × 0.15 × 0.12 mm

#### Data collection

Bruker APEXII CCD area-detector diffractometer
Absorption correction: multi-scan (*SADABS*; Bruker, 2001 ▶) *T*_min_ = 0.695, *T*_max_ = 0.807
19615 measured reflections
8987 independent reflections
6765 reflections with *I* > 2σ(*I*)
*R*_int_ = 0.036

#### Refinement

*R*[*F* ^2^ > 2σ(*F* ^2^)] = 0.037
*wR*(*F* ^2^) = 0.089
*S* = 1.00
8987 reflections
433 parameters
H-atom parameters constrained
Δρ_max_ = 0.59 e Å^−3^
Δρ_min_ = −0.56 e Å^−3^

### 

Data collection: *APEX2* (Bruker, 2007 ▶); cell refinement: *APEX2*; data reduction: *APEX2*; program(s) used to solve structure: *SHELXTL* (Sheldrick, 2008 ▶); program(s) used to refine structure: *SHELXTL*; molecular graphics: *SHELXTL*; software used to prepare material for publication: *SHELXTL*.

## Supplementary Material

Crystal structure: contains datablocks I, global. DOI: 10.1107/S1600536808003930/om2211sup1.cif

Structure factors: contains datablocks I. DOI: 10.1107/S1600536808003930/om2211Isup2.hkl

Additional supplementary materials:  crystallographic information; 3D view; checkCIF report

## Figures and Tables

**Table:** 

Zn1—O13	1.9541 (14)
Zn1—O1*W*	1.9599 (14)
Zn1—N1	2.0157 (17)
Zn1—O1	2.0955 (14)
Zn1—O3	2.4440 (15)
Zn2—N2	1.9965 (17)
Zn2—N3	2.0143 (17)
Zn2—O11	2.0715 (14)
Zn2—O8	2.2311 (15)
Zn2—O6	2.2320 (15)
Zn2—O13	2.3857 (14)

**Table:** 

O13—Zn1—O1	101.14 (6)
O1—Zn1—O3	151.36 (5)
N2—Zn2—N3	162.44 (7)
O11—Zn2—O6	90.99 (6)
O8—Zn2—O6	152.06 (5)
O11—Zn2—O13	151.16 (5)
O8—Zn2—O13	86.07 (5)

**Table 2:** Hydrogen-bond geometry (Å, °)

*D*—H⋯*A*	*D*—H	H⋯*A*	*D*⋯*A*	*D*—H⋯*A*
N4—H4*A*⋯O6^i^	0.88	2.17	2.894 (2)	139
N4—H4*B*⋯O14^ii^	0.88	1.95	2.810 (2)	164
N5—H5*A*⋯O1*W*^ii^	0.88	2.29	3.074 (2)	149
N5—H5*B*⋯O12^iii^	0.88	2.18	2.949 (2)	146
N6—H6*A*⋯O3	0.88	2.08	2.901 (2)	156
N6—H6*B*⋯O12^iii^	0.88	2.09	2.882 (2)	150
O4—H4*C*⋯O3*W*	0.84	1.75	2.586 (2)	170
O5—H5*C*⋯O9^iv^	0.84	1.78	2.596 (2)	163
O10—H10*A*⋯O2^v^	0.84	1.78	2.615 (2)	171
O15—H15⋯O1^vi^	0.84	1.87	2.637 (2)	152
O1*W*—H1⋯O2*W*^vii^	0.85	1.79	2.642 (2)	175
O1*W*—H2⋯O7^i^	0.85	1.76	2.609 (2)	178
O2*W*—H3⋯O8	0.85	2.07	2.912 (2)	171
O2*W*—H4⋯O3*W*	0.85	2.02	2.827 (2)	158
O3*W*—H5⋯O11^i^	0.85	1.78	2.622 (2)	170
O3*W*—H6⋯O10^iv^	0.85	2.59	3.344 (2)	148
C4—H4*D*⋯O9^iv^	0.95	2.30	2.993 (2)	129
C9—H9*A*⋯O2^v^	0.95	2.37	3.046 (3)	128

Symmetry codes: (i) 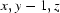; (ii) 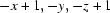; (iii) 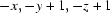; (iv) 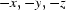; (v) 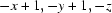; (vi) 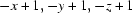; (vii) 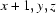.

**Fig. 1.**
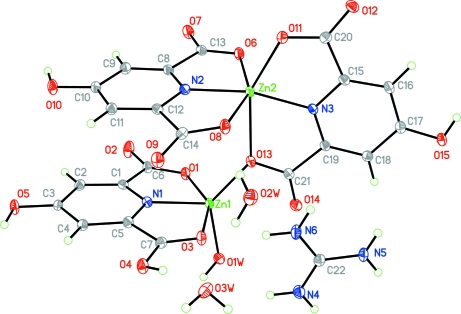
The molecular structure of the title compound, showing the atom numbering scheme. Displacement ellipsoids are drawn at the 50% probability level.

**Fig. 2.**
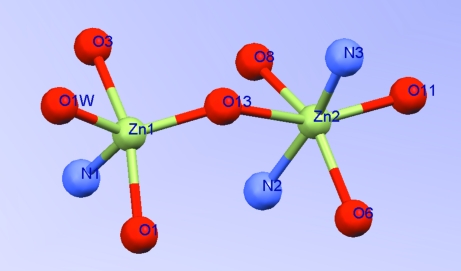
Coordination polyhedron around two metallic centers in the asymmetric unit.

**Fig. 3.**
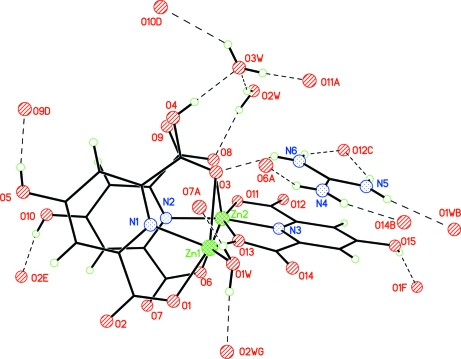
Hydrogen bondings in the title compound are shown with dashed lines. Symmetry codes to generate equivalent atoms are A: x, y - 1, z; B: -x + 1, -y, -z + 1; C: -x, -y + 1, -z + 1; D: -x, -y, -z; E: -x + 1, -y + 1, -z; F: -x + 1, -y + 1, -z + 1; G: x + 1, y, z.

**Fig. 4.**
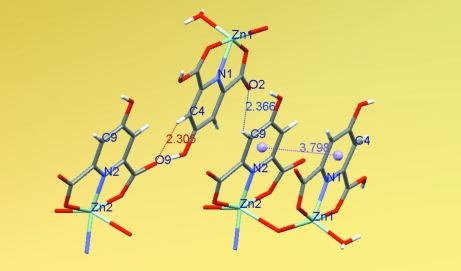
The two C–H···O bond lengths with symmetry codes of (-x, -y, -z) and (-x + 1, -y + 1, -z), and π-π distances between N1/C1—C5 and N2/C8—C12 rings of the asymmetric unit.

**Fig. 5.**
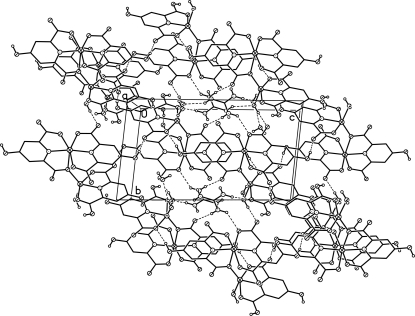
The crystal packing of the compound as viewed down a. Hydrogen bonds are shown as dashed lines.

**Fig. 6.**
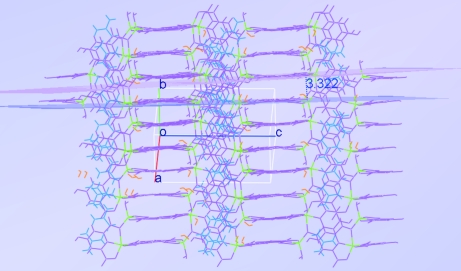
The packing of compound shows a type of channels between the layers of (hypydc)2- rings with the distance of ~3.3 Å.

## supplementary crystallographic information

### Comment

After the synthesis of proton transfer ion pairs with the formulae of (GH)(hypydcH) (Moghimi, Aghabozorg, Soleimannejad *et al.*, 2005) and (GH)(hypydcH).H_2_O (Moghimi, Aghabozorg, Sheshmani *et al.*, 2005), the first metallic compound related to them formulated as (GH)_2_[Ni(hypydc)_2_] .2H_2_O was synthesized (Aghabozorg, Motyeian *et al.*, 2008). Slightly different, the title compound is another metallic compound related to the mentioned ion pairs.

The molecular structure of the compound is shown in Fig. 1. A s it can be observed, the anionic complex involves two Zn^II^ atoms with different coordination modes including penta and hexa-coordination. One Zn^II^ is coordinated to two (hypydc)^2-^ groups, one of which is bridged to the second Zn^II^ which is also coordinated to an (hypydcH)^- ^group and a water molecule. The coordination polyhedron around Zn1 is a distorted trigonal bipyramid, while that of Zn2 is a distorted octahedron (Fig. 2). It is notable that the –COOH of the (hypydcH)^-^group is coordinated to Zn1 *via *its carbonyl O3 atom, while the –COO^-^ group is coordinated to Zn1 through its O1 atom, as usual. This is shown by essentially different C7–O3 (1.229 (2) Å) and C6–O1 (1.273 (2) Å) bond lengths. Moreover, (GH)^+^ as counter ion and two uncoordinated water molecules are incorporated in the structure. Investigating the angles of (GH)^+ ^shows that the sum of all three angles around C22 (120.63 (19), 120.05 (19) and 119.30 (19)°) confirms the coplanarity of the three bonds. In addition, since the three bonds of (GH)^+ ^are almost the same (N4–C22, 1.325 (3); N5–C22, 1.328 (3) and N6–C22, 1.327 (3) Å), it can be concluded that the positive charge is delocalized on the counter ion.

By comparison, the bond length of Zn1–O1 (2.0955 (14) Å) is obviously shorter than Zn1–O3 (2.4440 (15) Å). This is due to that O3 is of unprotonated –COOH group, but O1 is belonged to the deprotonated carboxylate group. Thus, Zn1–O3 is longer than the Zn1–O1 bond. This resembles to (pydaH)[Zn(pydc)(pydcH)]. 3H_2_O (pyda: pyridine-2,6-diamine, pydcH_2_: pyridine-2,6-dicarboxylic acid) complex in which the carbonyl oxygen atom of –COOH group forms a longer bond to metallic center (Ranjbar *et al.*, 2002). Also, the length of Zn2–O13 is longer than Zn2–O11, *i.e. *the bridge O13 atom lies further to Zn2, this is similar to polymeric (GH)[Bi(pydc)(H_2_O)] complex in which the bridge oxygen atom forms a longer bond to Bi^III ^atom (Sharif *et al.*, 2007). Moreover, the lengths of Zn1–O13 (1.9541 (14) Å) and Zn2–O13 (2.3857 (14) Å) are significantly different. As the O13–Zn2–O8–C14 (-83.49 (14)°) and O13–Zn2–O6–C13 (86.01 (14)°) torsion angles and O6–Zn2–O11 (90.99 (6)°) and O8–Zn2–O13 (86.07 (5)°) bond angles show, the two (hypydc)^2-^ rings coordinated to Zn2 are almost perpendicular.

The bond angles of Zn2 to four oxygen atoms of carboxylate groups show that they are oriented on a flattened tetrahedral arrangement around the metallic center (Table 1).

As shown in Fig. 3, there are plenty of hydrogen bonds of type O–H···O and N–H···O ranging from 2.586 (2) to 3.344 (2) Å, and C–H···O bonds with 2.993 (2) and 3.046 (3) Å lengths between the fragments (Table 2). Also, there is a weak π-π interaction with distance of 3.798 (1) Å between the aromatic rings (Fig. 4). The crystal packing of the compoud is shown in Fig. 5. A s illustrated in Fig. 6, there are a type of channels produced by aromatic rings of the structure with the distance of ~3.3 Å.

### Experimental

An aqueous solution of 200 mg guanidine hydrochloride (2 mmol) with 80 mg sodium hydroxide (2 mmol) was prepared. After stirring the obtained suspension, an aqueous solution of 291 mg ZnSO_4_^.^7H_2_O (1 mmol) and 360 mg 4-hydroxypyridine-2,6-dicarboxylic acid (2 mmol) was added to it. The mixture with the volume of 50 ml was heated and boiled for 2 h. Colorless crystals were obtained by slow cooling during three days.

### Refinement

The positions of hydrogen atoms on amino and hydroxo groups and water molecules were located from the difference Fourier syntheses and normalized to 0.88, 0.84 and 0.85%A distances, respectively. All hydrogen atom positions were refined in isotropic approximation in riding model with with the *U*_iso_(H) parameters equal to 1.2(N) for amino, 1.2Ueq(O) for hydroxo groups, to 1.2Ueq(C) for all carbon atoms and to 1.5Ueq(O) for water molecules where *U*_eq_(C) and *U*_eq_(O) are the equivalent thermal parameters of the atoms to which corresponding H atoms are bonded.

### Crystal data

**Table:**

(CH_6_N_3_)[Zn_2_(C_7_H_3_N_1_O_5_)_2_(C_7_H_4_N_1_O_5_)(H_2_O)]·2H_2_O	*Z* = 2
*M**_r_* = 789.20	*F*_000_ = 800
Triclinic, *P*1	*D*_x_ = 1.933 Mg m^−^^3^
Hall symbol: -P 1	Mo *K*α radiation λ = 0.71073 Å
*a* = 9.1077 (12) Å	Cell parameters from 540 reflections
*b* = 9.2900 (12) Å	θ = 2.8–27.2º
*c* = 16.347 (3) Å	µ = 1.87 mm^−^^1^
α = 96.018 (4)º	*T* = 100 (2) K
β = 99.229 (4)º	Prism, colourless
γ = 91.760 (7)º	0.21 × 0.15 × 0.12 mm
*V* = 1356.1 (3) Å^3^	

### Data collection

**Table:**

Bruker APEXII CCD area-detector diffractometer	8987 independent reflections
Radiation source: fine-focus sealed tube	6765 reflections with *I* > 2σ(*I*)
Monochromator: graphite	*R*_int_ = 0.036
*T* = 100(2) K	θ_max_ = 31.5º
ω scans	θ_min_ = 2.3º
Absorption correction: multi-scan(SADABS; Bruker, 2001)	*h* = −13→13
*T*_min_ = 0.695, *T*_max_ = 0.807	*k* = −13→13
19615 measured reflections	*l* = −23→24

### Refinement

**Table:**

Refinement on *F*^2^	Secondary atom site location: difference Fourier map
Least-squares matrix: full	Hydrogen site location: mixed
*R*[*F*^2^ > 2σ(*F*^2^)] = 0.037	H-atom parameters constrained
*wR*(*F*^2^) = 0.089	*w* = 1/[σ^2^(*F*_o_^2^) + (0.042*P*)^2^] where *P* = (*F*_o_^2^ + 2*F*_c_^2^)/3
*S* = 1.00	(Δ/σ)_max_ = 0.001
8987 reflections	Δρ_max_ = 0.59 e Å^−^^3^
433 parameters	Δρ_min_ = −0.56 e Å^−^^3^
Primary atom site location: structure-invariant direct methods	Extinction correction: none

### Special details

**Table:**

Geometry. All e.s.d.'s (except the e.s.d. in the dihedral angle between two l.s. planes) are estimated using the full covariance matrix. The cell e.s.d.'s are taken into account individually in the estimation of e.s.d.'s in distances, angles and torsion angles; correlations between e.s.d.'s in cell parameters are only used when they are defined by crystal symmetry. An approximate (isotropic) treatment of cell e.s.d.'s is used for estimating e.s.d.'s involving l.s. planes.
Refinement. Refinement of *F*^2^ against ALL reflections. The weighted *R*-factor *wR* and goodness of fit *S* are based on *F*^2^, conventional *R*-factors *R* are based on *F*, with *F* set to zero for negative *F*^2^. The threshold expression of *F*^2^ > σ(*F*^2^) is used only for calculating *R*-factors(gt) *etc*. and is not relevant to the choice of reflections for refinement. *R*-factors based on *F*^2^ are statistically about twice as large as those based on *F*, and *R*- factors based on ALL data will be even larger.

### Fractional atomic coordinates and isotropic or equivalent isotropic displacement parameters (Å^2^)

**Table:**

	*x*	*y*	*z*	*U*_iso_*/*U*_eq_	
Zn1	0.43557 (3)	0.16470 (2)	0.258415 (14)	0.01093 (6)	
Zn2	0.18238 (3)	0.50031 (2)	0.310910 (14)	0.01149 (6)	
N1	0.36197 (18)	0.12238 (18)	0.13492 (10)	0.0107 (3)	
N2	0.18759 (19)	0.47854 (18)	0.18862 (10)	0.0110 (3)	
N3	0.23352 (18)	0.48751 (17)	0.43438 (10)	0.0099 (3)	
N4	0.3338 (2)	−0.10238 (19)	0.43263 (11)	0.0157 (4)	
H4A	0.3274	−0.1284	0.3788	0.019*	
H4B	0.3992	−0.1414	0.4684	0.019*	
N5	0.2568 (2)	0.03721 (19)	0.54093 (11)	0.0156 (4)	
H5A	0.3235	−0.0004	0.5767	0.019*	
H5B	0.1978	0.1023	0.5589	0.019*	
N6	0.1460 (2)	0.0554 (2)	0.40657 (11)	0.0148 (4)	
H6A	0.1387	0.0300	0.3526	0.018*	
H6B	0.0875	0.1205	0.4252	0.018*	
O1	0.59008 (16)	0.29141 (15)	0.21141 (9)	0.0137 (3)	
O2	0.63792 (18)	0.36048 (18)	0.09069 (9)	0.0215 (3)	
O3	0.21305 (16)	0.00289 (16)	0.23874 (9)	0.0145 (3)	
O4	0.07106 (17)	−0.13769 (16)	0.13266 (9)	0.0153 (3)	
H4C	0.0149	−0.1491	0.1678	0.018*	
O5	0.27628 (16)	0.02110 (16)	−0.11860 (9)	0.0143 (3)	
H5C	0.2173	−0.0520	−0.1315	0.017*	
O6	0.34222 (17)	0.67780 (16)	0.29445 (9)	0.0146 (3)	
O7	0.44783 (17)	0.76832 (16)	0.19381 (9)	0.0172 (3)	
O8	0.00950 (16)	0.32407 (16)	0.26076 (9)	0.0143 (3)	
O9	−0.05698 (17)	0.17203 (16)	0.14388 (9)	0.0159 (3)	
O10	0.19172 (17)	0.42385 (16)	−0.06347 (9)	0.0171 (3)	
H10A	0.2427	0.4907	−0.0779	0.021*	
O11	0.04286 (16)	0.64962 (15)	0.35657 (9)	0.0135 (3)	
O12	−0.01195 (16)	0.76190 (15)	0.47515 (9)	0.0147 (3)	
O13	0.35192 (16)	0.31130 (15)	0.32996 (9)	0.0129 (3)	
O14	0.49768 (17)	0.21787 (16)	0.43224 (9)	0.0166 (3)	
O15	0.31816 (17)	0.47150 (16)	0.68773 (9)	0.0160 (3)	
H15	0.3166	0.5532	0.7150	0.019*	
C1	0.4418 (2)	0.1863 (2)	0.08551 (12)	0.0107 (4)	
C2	0.4131 (2)	0.1565 (2)	−0.00030 (12)	0.0122 (4)	
H2A	0.4696	0.2048	−0.0342	0.015*	
C3	0.2999 (2)	0.0541 (2)	−0.03617 (12)	0.0110 (4)	
C4	0.2169 (2)	−0.0136 (2)	0.01602 (12)	0.0115 (4)	
H4D	0.1386	−0.0835	−0.0065	0.014*	
C5	0.2529 (2)	0.0247 (2)	0.10110 (12)	0.0112 (4)	
C6	0.5679 (2)	0.2891 (2)	0.13240 (13)	0.0127 (4)	
C7	0.1755 (2)	−0.0385 (2)	0.16419 (12)	0.0118 (4)	
C8	0.2775 (2)	0.5651 (2)	0.15616 (12)	0.0116 (4)	
C9	0.2842 (2)	0.5519 (2)	0.07219 (13)	0.0136 (4)	
H9A	0.3492	0.6146	0.0506	0.016*	
C10	0.1938 (2)	0.4448 (2)	0.01905 (12)	0.0127 (4)	
C11	0.1009 (2)	0.3538 (2)	0.05356 (12)	0.0127 (4)	
H11A	0.0391	0.2794	0.0193	0.015*	
C12	0.1010 (2)	0.3745 (2)	0.13860 (12)	0.0109 (4)	
C13	0.3656 (2)	0.6799 (2)	0.22034 (13)	0.0124 (4)	
C14	0.0089 (2)	0.2824 (2)	0.18459 (12)	0.0118 (4)	
C15	0.1664 (2)	0.5794 (2)	0.48423 (12)	0.0109 (4)	
C16	0.1924 (2)	0.5833 (2)	0.57028 (12)	0.0123 (4)	
H16A	0.1455	0.6509	0.6041	0.015*	
C17	0.2895 (2)	0.4848 (2)	0.60600 (12)	0.0123 (4)	
C18	0.3603 (2)	0.3900 (2)	0.55377 (12)	0.0116 (4)	
H18A	0.4279	0.3230	0.5764	0.014*	
C19	0.3294 (2)	0.3963 (2)	0.46898 (12)	0.0107 (4)	
C20	0.0565 (2)	0.6734 (2)	0.43629 (12)	0.0114 (4)	
C21	0.4003 (2)	0.2998 (2)	0.40786 (12)	0.0106 (4)	
C22	0.2451 (2)	−0.0043 (2)	0.45983 (13)	0.0125 (4)	
O1W	0.55695 (16)	0.00217 (15)	0.28937 (9)	0.0130 (3)	
H1	0.6477	0.0287	0.2913	0.020*	
H2	0.5227	−0.0755	0.2593	0.020*	
O2W	−0.15742 (16)	0.07809 (17)	0.30343 (9)	0.0166 (3)	
H3	−0.1151	0.1488	0.2859	0.025*	
H4	−0.1159	0.0052	0.2830	0.025*	
O3W	−0.09191 (17)	−0.20352 (17)	0.24040 (9)	0.0180 (3)	
H5	−0.0481	−0.2423	0.2819	0.027*	
H6	−0.1443	−0.2697	0.2077	0.027*	

### Atomic displacement parameters (Å^2^)

**Table:**

	*U*^11^	*U*^22^	*U*^33^	*U*^12^	*U*^13^	*U*^23^
Zn1	0.01283 (12)	0.01187 (11)	0.00817 (11)	0.00039 (9)	0.00278 (8)	0.00002 (8)
Zn2	0.01472 (12)	0.01176 (11)	0.00832 (11)	0.00042 (9)	0.00333 (9)	0.00050 (8)
N1	0.0103 (8)	0.0110 (8)	0.0105 (8)	0.0004 (6)	0.0018 (6)	0.0002 (6)
N2	0.0132 (8)	0.0094 (7)	0.0110 (8)	−0.0014 (6)	0.0041 (6)	0.0017 (6)
N3	0.0107 (8)	0.0092 (7)	0.0098 (8)	−0.0002 (6)	0.0029 (6)	−0.0007 (6)
N4	0.0170 (9)	0.0171 (9)	0.0126 (8)	0.0048 (7)	0.0011 (7)	0.0004 (7)
N5	0.0209 (9)	0.0180 (9)	0.0080 (8)	0.0026 (7)	0.0023 (7)	0.0011 (6)
N6	0.0148 (8)	0.0211 (9)	0.0095 (8)	0.0057 (7)	0.0033 (6)	0.0023 (7)
O1	0.0154 (7)	0.0155 (7)	0.0095 (7)	−0.0029 (6)	0.0019 (5)	−0.0003 (5)
O2	0.0243 (9)	0.0271 (9)	0.0127 (7)	−0.0133 (7)	0.0042 (6)	0.0033 (6)
O3	0.0164 (7)	0.0181 (7)	0.0085 (7)	−0.0015 (6)	0.0024 (5)	−0.0003 (5)
O4	0.0176 (7)	0.0176 (7)	0.0111 (7)	−0.0068 (6)	0.0056 (6)	0.0004 (6)
O5	0.0154 (7)	0.0182 (7)	0.0081 (7)	−0.0071 (6)	0.0014 (5)	−0.0012 (5)
O6	0.0199 (8)	0.0138 (7)	0.0102 (7)	−0.0022 (6)	0.0049 (6)	0.0000 (5)
O7	0.0232 (8)	0.0137 (7)	0.0148 (7)	−0.0068 (6)	0.0065 (6)	−0.0011 (6)
O8	0.0166 (7)	0.0168 (7)	0.0100 (7)	−0.0036 (6)	0.0050 (6)	0.0004 (5)
O9	0.0170 (7)	0.0162 (7)	0.0138 (7)	−0.0068 (6)	0.0031 (6)	−0.0002 (6)
O10	0.0250 (8)	0.0169 (7)	0.0093 (7)	−0.0083 (6)	0.0050 (6)	0.0002 (6)
O11	0.0155 (7)	0.0145 (7)	0.0111 (7)	0.0023 (6)	0.0035 (5)	0.0018 (5)
O12	0.0151 (7)	0.0136 (7)	0.0162 (7)	0.0032 (6)	0.0059 (6)	0.0002 (6)
O13	0.0159 (7)	0.0131 (7)	0.0101 (7)	0.0018 (6)	0.0043 (5)	−0.0002 (5)
O14	0.0179 (8)	0.0169 (7)	0.0158 (7)	0.0067 (6)	0.0047 (6)	0.0009 (6)
O15	0.0227 (8)	0.0160 (7)	0.0081 (7)	0.0024 (6)	0.0005 (6)	−0.0016 (5)
C1	0.0109 (9)	0.0095 (8)	0.0120 (9)	0.0006 (7)	0.0032 (7)	0.0007 (7)
C2	0.0126 (9)	0.0133 (9)	0.0114 (9)	0.0010 (7)	0.0031 (7)	0.0029 (7)
C3	0.0131 (9)	0.0115 (9)	0.0084 (9)	0.0020 (7)	0.0015 (7)	0.0010 (7)
C4	0.0117 (9)	0.0112 (9)	0.0113 (9)	−0.0028 (7)	0.0012 (7)	0.0012 (7)
C5	0.0110 (9)	0.0122 (9)	0.0110 (9)	0.0000 (7)	0.0036 (7)	0.0014 (7)
C6	0.0136 (9)	0.0135 (9)	0.0110 (9)	−0.0022 (7)	0.0032 (7)	0.0000 (7)
C7	0.0116 (9)	0.0124 (9)	0.0124 (9)	0.0010 (7)	0.0039 (7)	0.0022 (7)
C8	0.0143 (9)	0.0091 (9)	0.0116 (9)	−0.0008 (7)	0.0031 (7)	0.0005 (7)
C9	0.0163 (10)	0.0125 (9)	0.0122 (9)	−0.0027 (8)	0.0033 (8)	0.0017 (7)
C10	0.0160 (10)	0.0124 (9)	0.0101 (9)	0.0010 (8)	0.0032 (7)	0.0014 (7)
C11	0.0145 (9)	0.0121 (9)	0.0113 (9)	−0.0012 (7)	0.0016 (7)	0.0010 (7)
C12	0.0110 (9)	0.0091 (8)	0.0123 (9)	−0.0012 (7)	0.0017 (7)	0.0005 (7)
C13	0.0156 (10)	0.0090 (8)	0.0125 (9)	−0.0011 (7)	0.0024 (7)	0.0003 (7)
C14	0.0105 (9)	0.0129 (9)	0.0122 (9)	0.0000 (7)	0.0018 (7)	0.0027 (7)
C15	0.0112 (9)	0.0089 (8)	0.0127 (9)	−0.0016 (7)	0.0035 (7)	−0.0002 (7)
C16	0.0136 (9)	0.0120 (9)	0.0111 (9)	−0.0016 (7)	0.0041 (7)	−0.0016 (7)
C17	0.0122 (9)	0.0125 (9)	0.0116 (9)	−0.0033 (7)	0.0024 (7)	−0.0006 (7)
C18	0.0121 (9)	0.0109 (9)	0.0115 (9)	−0.0014 (7)	0.0013 (7)	0.0003 (7)
C19	0.0116 (9)	0.0094 (8)	0.0112 (9)	−0.0014 (7)	0.0036 (7)	−0.0002 (7)
C20	0.0110 (9)	0.0101 (9)	0.0136 (9)	−0.0025 (7)	0.0032 (7)	0.0024 (7)
C21	0.0095 (9)	0.0106 (9)	0.0121 (9)	−0.0022 (7)	0.0044 (7)	0.0001 (7)
C22	0.0124 (9)	0.0128 (9)	0.0125 (9)	−0.0026 (7)	0.0029 (7)	0.0018 (7)
O1W	0.0127 (7)	0.0121 (7)	0.0132 (7)	−0.0014 (5)	0.0010 (5)	−0.0009 (5)
O2W	0.0159 (7)	0.0188 (8)	0.0158 (7)	−0.0018 (6)	0.0040 (6)	0.0032 (6)
O3W	0.0200 (8)	0.0199 (8)	0.0151 (7)	0.0022 (6)	0.0030 (6)	0.0069 (6)

### Geometric parameters (Å, °)

**Table:**

Zn1—O13	1.9541 (14)	O10—H10A	0.8400
Zn1—O1W	1.9599 (14)	O11—C20	1.284 (2)
Zn1—N1	2.0157 (17)	O12—C20	1.231 (2)
Zn1—O1	2.0955 (14)	O13—C21	1.296 (2)
Zn1—O3	2.4440 (15)	O14—C21	1.232 (2)
Zn2—N2	1.9965 (17)	O15—C17	1.339 (2)
Zn2—N3	2.0143 (17)	O15—H15	0.8400
Zn2—O11	2.0715 (14)	C1—C2	1.382 (3)
Zn2—O8	2.2311 (15)	C1—C6	1.522 (3)
Zn2—O6	2.2320 (15)	C2—C3	1.393 (3)
Zn2—O13	2.3857 (14)	C2—H2A	0.9500
N1—C5	1.334 (3)	C3—C4	1.409 (3)
N1—C1	1.340 (3)	C4—C5	1.383 (3)
N2—C8	1.337 (3)	C4—H4D	0.9500
N2—C12	1.344 (2)	C5—C7	1.497 (3)
N3—C19	1.337 (3)	C8—C9	1.377 (3)
N3—C15	1.346 (2)	C8—C13	1.524 (3)
N4—C22	1.325 (3)	C9—C10	1.401 (3)
N4—H4A	0.8800	C9—H9A	0.9500
N4—H4B	0.8800	C10—C11	1.399 (3)
N5—C22	1.328 (3)	C11—C12	1.383 (3)
N5—H5A	0.8800	C11—H11A	0.9500
N5—H5B	0.8800	C12—C14	1.515 (3)
N6—C22	1.327 (3)	C15—C16	1.385 (3)
N6—H6A	0.8800	C15—C20	1.525 (3)
N6—H6B	0.8800	C16—C17	1.399 (3)
O1—C6	1.273 (2)	C16—H16A	0.9500
O2—C6	1.233 (2)	C17—C18	1.405 (3)
O3—C7	1.229 (2)	C18—C19	1.377 (3)
O4—C7	1.307 (2)	C18—H18A	0.9500
O4—H4C	0.8400	C19—C21	1.507 (3)
O5—C3	1.331 (2)	O1W—H1	0.8500
O5—H5C	0.8400	O1W—H2	0.8500
O6—C13	1.265 (2)	O2W—H3	0.8501
O7—C13	1.251 (2)	O2W—H4	0.8499
O8—C14	1.264 (2)	O3W—H5	0.8499
O9—C14	1.243 (2)	O3W—H6	0.8500
O10—C10	1.340 (2)		
			
O13—Zn1—O1W	129.21 (6)	C2—C3—C4	119.01 (18)
O13—Zn1—N1	123.11 (6)	C5—C4—C3	117.96 (18)
O1W—Zn1—N1	105.76 (6)	C5—C4—H4D	121.0
O13—Zn1—O1	101.14 (6)	C3—C4—H4D	121.0
O1W—Zn1—O1	100.48 (6)	N1—C5—C4	122.62 (18)
N1—Zn1—O1	79.40 (6)	N1—C5—C7	113.35 (17)
O13—Zn1—O3	93.10 (6)	C4—C5—C7	124.03 (18)
O1W—Zn1—O3	89.12 (6)	O2—C6—O1	126.81 (19)
N1—Zn1—O3	72.01 (6)	O2—C6—C1	117.40 (18)
O1—Zn1—O3	151.36 (5)	O1—C6—C1	115.79 (17)
N2—Zn2—N3	162.44 (7)	O3—C7—O4	125.56 (18)
N2—Zn2—O11	118.12 (6)	O3—C7—C5	119.99 (18)
N3—Zn2—O11	78.99 (6)	O4—C7—C5	114.45 (17)
N2—Zn2—O8	76.65 (6)	N2—C8—C9	122.00 (18)
N3—Zn2—O8	106.40 (6)	N2—C8—C13	113.48 (17)
O11—Zn2—O8	97.59 (6)	C9—C8—C13	124.49 (18)
N2—Zn2—O6	75.85 (6)	C8—C9—C10	119.03 (19)
N3—Zn2—O6	101.32 (6)	C8—C9—H9A	120.5
O11—Zn2—O6	90.99 (6)	C10—C9—H9A	120.5
O8—Zn2—O6	152.06 (5)	O10—C10—C11	117.85 (18)
N2—Zn2—O13	90.61 (6)	O10—C10—C9	123.52 (18)
N3—Zn2—O13	72.57 (6)	C11—C10—C9	118.63 (18)
O11—Zn2—O13	151.16 (5)	C12—C11—C10	118.62 (18)
O8—Zn2—O13	86.07 (5)	C12—C11—H11A	120.7
O6—Zn2—O13	99.08 (5)	C10—C11—H11A	120.7
C5—N1—C1	119.59 (17)	N2—C12—C11	122.00 (18)
C5—N1—Zn1	124.22 (13)	N2—C12—C14	113.65 (17)
C1—N1—Zn1	115.71 (13)	C11—C12—C14	124.34 (17)
C8—N2—C12	119.71 (17)	O7—C13—O6	127.34 (19)
C8—N2—Zn2	120.75 (13)	O7—C13—C8	116.96 (18)
C12—N2—Zn2	119.54 (13)	O6—C13—C8	115.64 (17)
C19—N3—C15	118.98 (17)	O9—C14—O8	126.80 (19)
C19—N3—Zn2	124.50 (13)	O9—C14—C12	116.56 (18)
C15—N3—Zn2	116.52 (13)	O8—C14—C12	116.62 (17)
C22—N4—H4A	120.0	N3—C15—C16	122.62 (19)
C22—N4—H4B	120.0	N3—C15—C20	113.29 (17)
H4A—N4—H4B	120.0	C16—C15—C20	124.07 (18)
C22—N5—H5A	120.0	C15—C16—C17	118.16 (18)
C22—N5—H5B	120.0	C15—C16—H16A	120.9
H5A—N5—H5B	120.0	C17—C16—H16A	120.9
C22—N6—H6A	120.0	O15—C17—C16	124.33 (18)
C22—N6—H6B	120.0	O15—C17—C18	116.65 (18)
H6A—N6—H6B	120.0	C16—C17—C18	119.00 (18)
C6—O1—Zn1	114.71 (12)	C19—C18—C17	118.50 (19)
C7—O3—Zn1	109.54 (13)	C19—C18—H18A	120.7
C7—O4—H4C	109.5	C17—C18—H18A	120.8
C3—O5—H5C	109.5	N3—C19—C18	122.71 (18)
C13—O6—Zn2	114.20 (12)	N3—C19—C21	114.77 (17)
C14—O8—Zn2	112.58 (12)	C18—C19—C21	122.52 (18)
C10—O10—H10A	109.5	O12—C20—O11	125.77 (19)
C20—O11—Zn2	116.08 (13)	O12—C20—C15	119.31 (18)
C21—O13—Zn1	110.63 (12)	O11—C20—C15	114.91 (17)
C21—O13—Zn2	112.70 (12)	O14—C21—O13	123.87 (18)
Zn1—O13—Zn2	136.65 (7)	O14—C21—C19	120.94 (18)
C17—O15—H15	109.5	O13—C21—C19	115.19 (17)
N1—C1—C2	122.10 (18)	N4—C22—N6	120.63 (19)
N1—C1—C6	114.11 (17)	N4—C22—N5	120.05 (19)
C2—C1—C6	123.76 (18)	N6—C22—N5	119.30 (19)
C1—C2—C3	118.71 (18)	Zn1—O1W—H1	107.7
C1—C2—H2A	120.6	Zn1—O1W—H2	110.3
C3—C2—H2A	120.6	H1—O1W—H2	118.8
O5—C3—C2	118.70 (18)	H3—O2W—H4	103.0
O5—C3—C4	122.27 (18)	H5—O3W—H6	107.7
			
O13—Zn1—N1—C5	−89.84 (17)	C2—C3—C4—C5	−0.4 (3)
O1W—Zn1—N1—C5	75.66 (17)	C1—N1—C5—C4	−0.5 (3)
O1—Zn1—N1—C5	173.68 (17)	Zn1—N1—C5—C4	−172.25 (15)
O3—Zn1—N1—C5	−8.11 (15)	C1—N1—C5—C7	179.16 (17)
O13—Zn1—N1—C1	98.09 (15)	Zn1—N1—C5—C7	7.4 (2)
O1W—Zn1—N1—C1	−96.40 (14)	C3—C4—C5—N1	0.0 (3)
O1—Zn1—N1—C1	1.62 (14)	C3—C4—C5—C7	−179.55 (18)
O3—Zn1—N1—C1	179.83 (15)	Zn1—O1—C6—O2	175.64 (18)
N3—Zn2—N2—C8	−80.4 (3)	Zn1—O1—C6—C1	−4.7 (2)
O11—Zn2—N2—C8	85.88 (16)	N1—C1—C6—O2	−174.20 (18)
O8—Zn2—N2—C8	177.43 (16)	C2—C1—C6—O2	7.8 (3)
O6—Zn2—N2—C8	2.45 (15)	N1—C1—C6—O1	6.1 (3)
O13—Zn2—N2—C8	−96.76 (15)	C2—C1—C6—O1	−171.92 (19)
N3—Zn2—N2—C12	98.9 (3)	Zn1—O3—C7—O4	172.31 (16)
O11—Zn2—N2—C12	−94.91 (15)	Zn1—O3—C7—C5	−7.0 (2)
O8—Zn2—N2—C12	−3.36 (14)	N1—C5—C7—O3	1.3 (3)
O6—Zn2—N2—C12	−178.34 (16)	C4—C5—C7—O3	−179.03 (19)
O13—Zn2—N2—C12	82.45 (15)	N1—C5—C7—O4	−178.08 (17)
N2—Zn2—N3—C19	−15.1 (3)	C4—C5—C7—O4	1.5 (3)
O11—Zn2—N3—C19	177.21 (16)	C12—N2—C8—C9	0.3 (3)
O8—Zn2—N3—C19	82.45 (16)	Zn2—N2—C8—C9	179.54 (15)
O6—Zn2—N3—C19	−93.99 (16)	C12—N2—C8—C13	178.52 (17)
O13—Zn2—N3—C19	2.08 (15)	Zn2—N2—C8—C13	−2.3 (2)
N2—Zn2—N3—C15	164.06 (19)	N2—C8—C9—C10	0.2 (3)
O11—Zn2—N3—C15	−3.60 (14)	C13—C8—C9—C10	−177.83 (19)
O8—Zn2—N3—C15	−98.36 (14)	C8—C9—C10—O10	179.67 (19)
O6—Zn2—N3—C15	85.20 (14)	C8—C9—C10—C11	−0.7 (3)
O13—Zn2—N3—C15	−178.73 (15)	O10—C10—C11—C12	−179.61 (18)
O13—Zn1—O1—C6	−120.04 (14)	C9—C10—C11—C12	0.7 (3)
O1W—Zn1—O1—C6	106.20 (14)	C8—N2—C12—C11	−0.3 (3)
N1—Zn1—O1—C6	1.94 (14)	Zn2—N2—C12—C11	−179.50 (15)
O3—Zn1—O1—C6	−1.6 (2)	C8—N2—C12—C14	178.22 (17)
O13—Zn1—O3—C7	131.73 (14)	Zn2—N2—C12—C14	−1.0 (2)
O1W—Zn1—O3—C7	−99.05 (14)	C10—C11—C12—N2	−0.2 (3)
N1—Zn1—O3—C7	7.85 (13)	C10—C11—C12—C14	−178.59 (18)
O1—Zn1—O3—C7	11.5 (2)	Zn2—O6—C13—O7	178.91 (18)
N2—Zn2—O6—C13	−2.32 (14)	Zn2—O6—C13—C8	1.9 (2)
N3—Zn2—O6—C13	159.91 (14)	N2—C8—C13—O7	−177.34 (18)
O11—Zn2—O6—C13	−121.12 (14)	C9—C8—C13—O7	0.8 (3)
O8—Zn2—O6—C13	−12.8 (2)	N2—C8—C13—O6	0.0 (3)
O13—Zn2—O6—C13	86.01 (14)	C9—C8—C13—O6	178.2 (2)
N2—Zn2—O8—C14	8.08 (14)	Zn2—O8—C14—O9	167.49 (17)
N3—Zn2—O8—C14	−154.02 (14)	Zn2—O8—C14—C12	−11.0 (2)
O11—Zn2—O8—C14	125.28 (14)	N2—C12—C14—O9	−170.05 (18)
O6—Zn2—O8—C14	18.5 (2)	C11—C12—C14—O9	8.4 (3)
O13—Zn2—O8—C14	−83.49 (14)	N2—C12—C14—O8	8.6 (3)
N2—Zn2—O11—C20	−171.55 (13)	C11—C12—C14—O8	−172.96 (19)
N3—Zn2—O11—C20	4.25 (14)	C19—N3—C15—C16	−0.1 (3)
O8—Zn2—O11—C20	109.57 (14)	Zn2—N3—C15—C16	−179.36 (15)
O6—Zn2—O11—C20	−97.09 (14)	C19—N3—C15—C20	−178.17 (17)
O13—Zn2—O11—C20	13.9 (2)	Zn2—N3—C15—C20	2.6 (2)
O1W—Zn1—O13—C21	7.05 (16)	N3—C15—C16—C17	−1.7 (3)
N1—Zn1—O13—C21	168.94 (12)	C20—C15—C16—C17	176.16 (18)
O1—Zn1—O13—C21	−106.53 (13)	C15—C16—C17—O15	−176.19 (18)
O3—Zn1—O13—C21	98.44 (13)	C15—C16—C17—C18	2.1 (3)
O1W—Zn1—O13—Zn2	−171.49 (8)	O15—C17—C18—C19	177.56 (18)
N1—Zn1—O13—Zn2	−9.60 (14)	C16—C17—C18—C19	−0.9 (3)
O1—Zn1—O13—Zn2	74.93 (11)	C15—N3—C19—C18	1.5 (3)
O3—Zn1—O13—Zn2	−80.10 (10)	Zn2—N3—C19—C18	−179.35 (14)
N2—Zn2—O13—C21	170.56 (13)	C15—N3—C19—C21	−179.06 (17)
N3—Zn2—O13—C21	−4.31 (13)	Zn2—N3—C19—C21	0.1 (2)
O11—Zn2—O13—C21	−14.27 (19)	C17—C18—C19—N3	−0.9 (3)
O8—Zn2—O13—C21	−112.87 (13)	C17—C18—C19—C21	179.63 (17)
O6—Zn2—O13—C21	94.79 (13)	Zn2—O11—C20—O12	177.04 (16)
N2—Zn2—O13—Zn1	−10.92 (11)	Zn2—O11—C20—C15	−4.1 (2)
N3—Zn2—O13—Zn1	174.20 (12)	N3—C15—C20—O12	−179.99 (18)
O11—Zn2—O13—Zn1	164.25 (9)	C16—C15—C20—O12	2.0 (3)
O8—Zn2—O13—Zn1	65.65 (10)	N3—C15—C20—O11	1.0 (2)
O6—Zn2—O13—Zn1	−86.69 (11)	C16—C15—C20—O11	−176.98 (18)
C5—N1—C1—C2	1.2 (3)	Zn1—O13—C21—O14	7.2 (2)
Zn1—N1—C1—C2	173.70 (15)	Zn2—O13—C21—O14	−173.85 (16)
C5—N1—C1—C6	−176.84 (17)	Zn1—O13—C21—C19	−173.27 (12)
Zn1—N1—C1—C6	−4.4 (2)	Zn2—O13—C21—C19	5.6 (2)
N1—C1—C2—C3	−1.5 (3)	N3—C19—C21—O14	175.23 (18)
C6—C1—C2—C3	176.35 (18)	C18—C19—C21—O14	−5.3 (3)
C1—C2—C3—O5	−177.33 (18)	N3—C19—C21—O13	−4.3 (2)
C1—C2—C3—C4	1.1 (3)	C18—C19—C21—O13	175.18 (18)
O5—C3—C4—C5	177.99 (18)		

### Hydrogen-bond geometry (Å, °)

**Table:**

*D*—H···*A*	*D*—H	H···*A*	*D*···*A*	*D*—H···*A*
N4—H4A···O6^i^	0.88	2.17	2.894 (2)	139
N4—H4B···O14^ii^	0.88	1.95	2.810 (2)	164
N5—H5A···O1W^ii^	0.88	2.29	3.074 (2)	149
N5—H5B···O12^iii^	0.88	2.18	2.949 (2)	146
N6—H6A···O3	0.88	2.08	2.901 (2)	156
N6—H6B···O12^iii^	0.88	2.09	2.882 (2)	150
O4—H4C···O3W	0.84	1.76	2.586 (2)	170
O5—H5C···O9^iv^	0.84	1.78	2.596 (2)	163
O10—H10A···O2^v^	0.84	1.78	2.615 (2)	171
O15—H15···O1^vi^	0.84	1.87	2.637 (2)	152
O1W—H1···O2W^vii^	0.85	1.79	2.642 (2)	175
O1W—H2···O7^i^	0.85	1.76	2.609 (2)	178
O2W—H3···O8	0.85	2.07	2.912 (2)	171
O2W—H4···O3W	0.85	2.02	2.827 (2)	158
O3W—H5···O11^i^	0.85	1.78	2.622 (2)	170
O3W—H6···O10^iv^	0.85	2.59	3.344 (2)	148
C4—H4D···O9^iv^	0.95	2.30	2.993 (2)	129
C9—H9A···O2^v^	0.95	2.37	3.046 (3)	128

Symmetry codes: (i) *x*, *y*−1, *z*; (ii) −*x*+1, −*y*, −*z*+1; (iii) −*x*, −*y*+1, −*z*+1;  (iv) −*x*, −*y*, −*z*; (v) −*x*+1, −*y*+1, −*z*; (vi) −*x*+1, −*y*+1, −*z*+1; (vii) *x*+1, *y*, *z*.
